# Improved Release and Metabolism of Flavonoids by Steered Fermentation Processes: A Review

**DOI:** 10.3390/ijms151119369

**Published:** 2014-10-24

**Authors:** Huynh Nguyen Thai, John Van Camp, Guy Smagghe, Katleen Raes

**Affiliations:** 1 Department of Industrial Biological Sciences, Faculty of Bioscience Engineering, Ghent University—Campus Kortrijk, Graaf Karel de Goedelaan 5, 8500 Kortrijk, Belgium; E-Mail: thainguyen.huynh@ugent.be; 2 Department of Food Safety and Food Quality, Faculty of Bioscience Engineering, Ghent University, Coupure Links 653, 9000 Ghent, Belgium; E-Mail: john.vancamp@ugent.be; 3 Department of Crop Protection, Faculty of Bioscience Engineering, Ghent University, Coupure Links 653, 9000 Ghent, Belgium; E-Mail: guy.smagghe@ugent.be; 4 Faculty of Food Technology, Ho Chi Minh City University of Food Industry, 140 Le Trong Tan, Tay Thanh Ward, Tan Phu District, 700000 Ho Chi Minh City, Vietnam

**Keywords:** fermentation, flavonoids, bioconversion, metabolism

## Abstract

This paper provides an overview on steered fermentation processes to release phenolic compounds from plant-based matrices, as well as on their potential application to convert phenolic compounds into unique metabolites. The ability of fermentation to improve the yield and to change the profile of phenolic compounds is mainly due to the release of bound phenolic compounds, as a consequence of the degradation of the cell wall structure by microbial enzymes produced during fermentation. Moreover, the microbial metabolism of phenolic compounds results in a large array of new metabolites through different bioconversion pathways such as glycosylation, deglycosylation, ring cleavage, methylation, glucuronidation and sulfate conjugation, depending on the microbial strains and substrates used. A whole range of metabolites is produced, however metabolic pathways related to the formation and bioactivities, and often quantification of the metabolites are highly underinvestigated. This strategy could have potential to produce extracts with a high-added value from plant-based matrices.

## 1. Introduction

Phenolic compounds are secondary metabolites produced in plants by the shikimic acid pathway. They consist of a variety of structures divided into a number of main groups including phenolic acids, flavonoids, lignans and stilbenes depending on the number of benzene rings as well as on their structure of the carbon skeleton [1]. Among these, flavonoids are the most widespread group of phenolic compounds and are further divided into different subclasses *i.e.*, chalcones, flavonols, flavones, flavanones, flavanols, isoflavones and anthocyanins, based on various substitution patterns on rings A and B as well as variations in ring C. The second most popular group of phenolic compounds are phenolic acids, which can be divided into two categories: hydroxybenzoic acids and hydroxycinnamic acids. The most common hydroxybenzoic acids are gallic acid, salicylic acid, vanillic acid, protocatechuic acid and *p*-hydroxybenzoic acid, while *p*-coumaric acid, caffeic acid, sinapic acid and ferulic acid are the best known hydroxycinnamic acids [2,3].

There are several methods including physical, physicochemical and chemical techniques to extract phenolic compounds from plant and food products such as cold pressing, supercritical fluid extraction and organic solvent extraction [4], ultrasound-assisted extraction [5] and microwave-assisted extraction [6]. The disadvantage of these techniques is their low extraction yield of bound phenolic compounds, as these are mostly linked to plant cell wall material through a –OH group (*O*-glycosides) or carbon-carbon bonds (*C*-glycosides) [7]. As a consequence, the application of a hydrolysis step prior to conventional extraction could be used to maximize the extraction yield. Among these, enzymatic treatments and/or fermentation are preferred instead of the utilization of chemical pretreatments (acids or alkaline) as the latter results in hazards and toxicological effects due to the use of chemical products, in negative effects on the environment, and of unwanted transformations of the extracted compounds [8].

It has already been demonstrated that phenolic compounds have a wide range of bioactivities including antioxidative, anticarcinogenic, anti-inflammatory properties which are mostly related to the potential health-promoting benefits against human health risks such as hypertension, obesity, cardiovascular diseases, diabetes and cancer [9,10,11]. Recently, more and more evidence is available indicating that the different classes and structures of phenolic compounds result in considerable variations in their bioactivities, bioaccessibility, bioavailability as well as their metabolism in the human gastrointestinal system [1]. Heim *et al.* [12] reported that the phenolic aglycones have a higher antioxidative activity than their glycosides. Aglycones can be effectively absorbed through the small intestine, while the glycosidic forms, due to their complex structures and large molecular weights, reach the large intestine and are metabolized by human gut microbiota into different more simplified metabolites, which can be later absorbed [1,13,14]. Currently, phase I/II metabolites of phenolic compounds such as deglucosides [15,16,17], sulfoconjugates [18,19] and glucuronides [20], can be obtained by a fermentation process. It is therefore interesting to use bacterial or fungal fermentation processes, which not only enhance the release of bound phenolic compounds from the plant cell walls, but also convert phenolic compounds into different metabolites, which can exert other bioactivities. In this paper, release of phenolic compounds indicates the phenolic compounds obtained in a soluble free form in the fermentation medium. This contributes to the production of extracts and food products with a high added value.

Although several reviews are available dealing with different aspects of phenolic compounds [3,7,21,22,23], as far as we now there is no review focusing on microbial conversion of phenolic compounds into new metabolites by a steered fermentation process, *i.e.*, by a controlled fermentation process adding a pure microbial strain. The aim of this paper is thus to provide an overview of the profile in phenolic compounds and their metabolites obtained by microbial fermentation from plant material with the final aim to obtain (purified) extracts rich in phenolic compounds. The microbial fermentation processes which occur during digestion fall outside the scope of this paper. The effect of microorganisms (bacteria, yeast and fungi) used in fermentation processes to release phenolic compounds from plant matrices in the fermentation media as well as the possible metabolic pathways of flavonoids (glycosylation, deglycosylation, ring cleavage, methylation, glucuronidation, and sulfate conjugation) to new conversion products are summarized and discussed.

## 2. The Release of Phenolic Compounds from Plant Matrices by Fermentation Processes

Phenolic compounds present in plants can be classified into free phenolic compounds found in the vacuoles of plant cells, and bound phenolic compounds linked to cell wall structure components (cellulose, hemicellulose, lignin, pectin and protein) through several covalent bounds [24,25,26]. Free phenolic compounds can be effectively extracted by conventional techniques, while several hydrolysis processes have been used to enhance the release of bound phenolics. Fermentation has been considered as one of the best processes to obtain extracts with a high quality and a high activity, using economically and environmental friendly techniques [3].

Several studies reported that fermentation influences the phenolic profile of extracts obtained from various plant sources or during the fermentation of plant sources. Table 1 summarizes the published papers between 2000 and 2014, available on the Web of Science, including the phenolic profile changes, only measured by chromatographic techniques, and obtained by a steered fermentation process (no spontaneous or natural fermentations). Vattem *et al.* [27,28] have found that solid-state fermentation of cranberry pomace using a food-grade fungus *Lentinus edodes* resulted in a maximum of a 49% increase in ellagic acid content after five days of incubation. Another study demonstrated that the phenolic acid profile in an ethanolic extract from oat fermented by three different filamentous fungi (*Aspergillus oryzae* var. *effuses*, *Aspergillus oryzae* and *Aspergillus niger*) at 25 °C for three days was remarkably improved in comparison with non-fermented oat [29]. Indeed, the study showed that fermentation of oat using *Aspergillus oryzae* var. *effuses* or *Aspergillus oryzae* increased the content of caffeic acid and ferulic acid in oat (*Avena sativa* L.) up to about 2.7- to three-fold and 5.5- to nine-fold, respectively, when compared to native oat. Fermentation with *Aspergillus oryzae* var. *effuses* also resulted in a more than 100% increase of chlorogenic and *p*-coumaric acids. In a recent study, Schmidt *et al.* [30] investigated the effect of solid-state fermentation by *Rhizopus oryzae* on the profile of phenolic acids derived from rice bran. The content of chlorogenic acid, *p*-hydroxybenzoic acid and vanillin significantly increased during fermentation. According to these authors, an incubation for 120 h at 30 °C with *Rhizopus oryzae* led to the most substantial increase in gallic acid and ferulic acid content, ranging from 3 and 33 mg/g dried weight in native bran to 155 and 765 mg/g dried weight in fermented bran, respectively.

**Table 1:** The effect of microbial fermentation on the increase in phenolic compounds from various plant-based foods.

Microorganism	Source	Phenolic profile	Reference
**Bacteria**			
*Bacillus pumilus*	Soybean	Gallic acid, catechin, epicatechin	[31]
*Bacillus subtilis*	Soybean	Chlorogenic acid, naringin	[32]
*Bacillus subtilis*	Cheonggukjang (soybean paste)	Daidzein, genistein	[33,34]
*Lactobacillus acidophilus*	Apple juice	Gallic acid	[35]
*Lactobacillus johnsonii*, *Lactobacillus reuteri*, *Lactobacillus acidophilus*	Whole grain barley, oat groat	Sinapic acid, caffeic acid, *p*-coumaric acid, ferulic acid	[36]
*Lactobacillus plantarum*	Cowpeas	Quercetin	[37]
*Lactobacillus plantarum*, *Lactobacillus delbrueckii* supsp. *lactis*	Soybean	Daidzein, genistein	[38]
**Yeast**			
*Saccharomyces cerevisiae*	Wheat bran	Syringic acid, *p*-coumaric acid, ferulic acid	[39]
**Fungi**			
*Aspergillus oryzae*, *Monascus purpureus*	Soybean	Daidzein, genistein	[40,41,42]
*Aspergillus oryzae* var. *effuses*, *Aspergillus oryzae*, *Aspergillus niger*	Oat (*Avena sativa* L.)	Chlorogenic acid, ferulic acid, *p*-coumaric acid, caffeic acid	[29]
*Aspergillus oryzae*	Green tea	Gallic acid, gallocatechin, epigallocatechin, epicatechin, 3-*p*-coumaroylquinic acid, kaempferol-rutinoside	[43]
*Lentinus edodes*	Cranberry pomace (*Vaccinium acrocarpon*)	Ellagic acid	[27,28]
*Rhizopus oryzae*	Rice bran	Gallic acid, ferulic acid, *p*-hydroxybenzoic acid, caffeic acid, chlorogenic acid, vanillin	[30]
*Rhizopus oligosporus*, *Rhizopus oryzae*	Black soybean	Daidzein, genistein	[44]

In addition to phenolic acids, the enhancement of the flavonoid content has also been observed in recent studies. Soybeans incubated with *Aspergillus oryzae* at 30 °C for 48 h resulted in a 23-fold increase in genistein aglycones when compared to the content found in unfermented soybean flour [40]. The amount of these aglycones was also found to be higher in solid-state fermentations of soybean with *Rhizopus* sp. [44] and *Monascus purpureus* [41] compared to unfermented soybeans. 

Similar to filamentous fungus, different food-graded lactic acid bacteria (LAB) and *Bacillus* spp. have been evaluated for their potential to release phenolic acids as well as flavonoids from plant sources such as soybean [31,32], apple [35] and cereals [36]. The fermentation with *Lactobacillus johnsonii*, *Lactobacillus reuteri* and *Lactobacillus acidophilus* showed a 20-fold increase in the content of total free phenolic acids in both barley and oat flour, compared to the unfermented sample, with the largest increase observed for free ferulic acid up to 39–56 µg/g dried weight depending on the strains used, while the amount of this compound found in unfermented samples was around 1 µg/g dried weight [36]. This study also exhibited that fermentation with *Lactobacillus johnsonii* had a much higher effect on the release of free phenolic acids than the other strains. A similar effect on the release of bound phenolic compounds was observed. Fermentation of grain barley with three LAB strains resulted in a significant increase of ferulic acid and *p*-coumaric acid which contributed to an increase in total content of bound phenolic acids by around 23%, compared to native grain barley. Also, enhancing the release of phenolic acids and flavanols was reported in a recent study [31], showing 2.8-fold, 7.6-fold and 4.5-fold increases in gallic acid, catechin and epicatechin, respectively after 60 h of fermentation with *Bacillus pumilus*. Soybean seeds fermented with *Bacillus subtilis* for three days yielded an increase in chlorogenic acid and naringin [32]. 

Not only fungi, LAB strains and *Bacillus* spp. have been used, but also yeast were screened for their improvement of the free phenolic profile. Moore *et al.* [39] reported that solid-state fermentation of wheat bran with *Saccharomyces cerevisiae* yielded a maximum increase of 48%, 51% and 333% in the content of soluble free *p*-coumaric, ferulic and syringic acid, respectively, compared to unfermented samples, while soluble vanillic acid decreased probably due to its conversion into other metabolites. 

However, a fermentation process does not exclusively increase all phenolic compounds. Also, a decrease in some components, e.g., deglycosylated, is observed, as they are metabolized into other forms having a lower toxicity towards microbial activity. Also type of microorganism, conditions of the fermentation process and fermentation time play a role herein [42,43,45].

**Figure 1:**
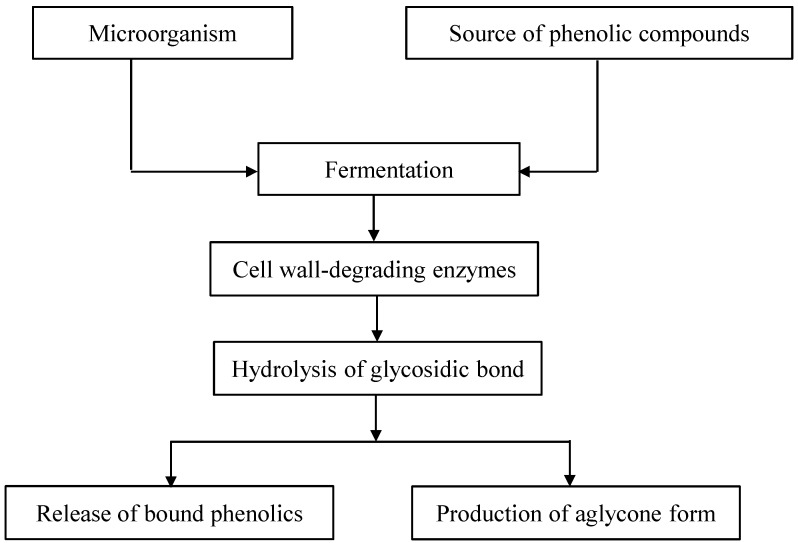
A schematic diagram of the release and bioconversion of phenolic compounds.

The change in the profile of phenolic compounds by the fermentation process is due to the action of cellulolytic, ligninolytic and pectinolytic enzymes, mainly produced during the growth of the microorganisms, as described in Figure 1. An overview of possible enzymes involved in the release of phenolic compounds, by breaking down the cell wall matrix and produced by the fermentation microorganisms is given in Table 2. These enzymes are known to be capable of completely breaking down the chemical components of plant cell walls, resulting in the hydrolysis of the ester bonds, which link phenolic compounds to the cell wall matrix, and in the oxidative degradation of lignin. As a consequence, the free phenolic compounds as well as bound forms are released more efficiently from the plant matrix. Among these enzymes, β-glucosidase has been widely reported as an enzyme responsible for catalyzing the hydrolysis of glycosidic linkages in alkyl and aryl-β-d-glucosides to release phenolic aglycone moieties. Vattem *et al.* [27] demonstrated that the increased release of the aglycone form of ellagic acid from cranberry pomace could be attributed to crude β-glucosidase produced, during solid-state fermentation by the food-grade fungi *Lentinus edodes*. Similarly, previous studies pointed out that esterases produced by filamentous fungi through solid-state fermentation of cereal sources, such as oat [29] and rice bran [30], caused an increase in the content of phenolic acids, such as ferulic acid, caffeic acid and *p*-coumaric acid.

The changes in phenolic profile were observed as a result of microbial fermentation of plant-based matrices. This confirms that the fermentation of plant substrates, both edible parts as well as agricultural by-products and food waste, with different microorganisms, including filamentous fungi, lactic acid bacteria, yeast, could be considered as a potential process to increase the release of phenolic compounds contributing to the production of extracts and food products with an added value.

## 3. Microbial Metabolism of Flavonoids during Fermentation

**Figure 2:**
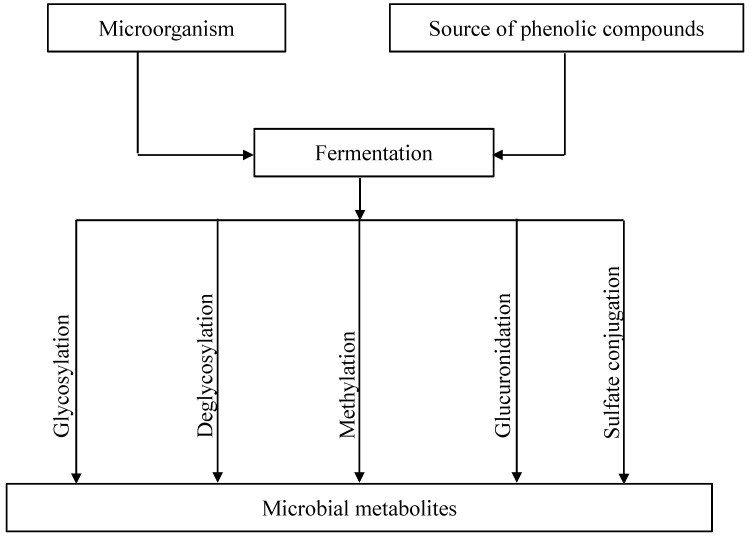
A schematic diagram of microbial conversion of phenolic compounds.

Various metabolic pathways of phenolic compounds by microbial fermentation are summarized in Figure 2. The studies dealing with the bioconversion of flavonoids into their metabolites by a controlled microbial fermentation are summarized in Table 3. However, it should be mentioned that not all of the studied microorganisms are food-graded ones. Besides, only wild-type microorganisms are included, possessing the characteristics to modify phenolic compounds. Although some recent studies are investigating genetically modified organisms to obtain higher conversion yields by expressing certain enzymes, these studies are not included in this paper. A more detailed discussion of the different processes is given below.

**Table 2:** Enzymes system produced by different microorganism strains to degrade the cell wall matrix.

Microorganisms	Species	Enzymes	References
Bacteria	*Lactobacillus lactis*	Esterase, decarboxylase	[46]
	*Lactobacillus plantarum*	β-Glucosidase, decarboxylase	[46]
	*Lactobacillus rhamnosus*	Cellulase, esterase, β-glucosidase	[46,47]
	*Bacillus cereus*	Cellulase, tannase	[46]
	*Bacillus subtilis*	Cellulase, β-glucanase	[46]
	*Bacillus thuringiensis*	Cellulase, tannase	[46]
Fungi	*Aspergillus awamori nakazawa*	Xylanase, α-l-arabinofuranosidase, feruloyl esterase	[48,49]
	*Aspergillus niger*	Cellulase, esterase, β-glucosidase, xylanase	[46,48,49,50]
	*Aspergillus oryzae*	Cellulase, β-glucosidase, xylanase, pectinase	[51]
	*Lentinus edodes*	Cellulase, β-glucosidase, xylanase, manganese peroxidase, laccase	[52,53]
	*Penicillium brasilianum*	Feruoylesterase	[54]
	*Pleurotus ostreatus*	Laccase, α-/β-glucosidase	[55,56]
	*Rhizopus oligosporus*	β-glucosidase, β-glucuronidase, xylanase	[57,58,59]
	*Phanerochaete chrysosporium*	β-Glucosidase, lignin peroxidases, manganese peroxidase, laccase	[60]
	*Rhizopus oryzae*	β-glucosidase, tannase, pectinase	[24]
Yeast	*Crytococcus flavus*	β-glucosidase, β-glucanase, esterase, xylanase	[46]
	*Rhodotorula glutimis*	β-Glucosidase	[46]
	*Sacharomyces cerevisiae*	β-Glucosidase, feruoylesterase	[46,61]
	*Wickerhamomyces anomalus*	β-Glucosidase, esterase	[62]

**Table 3:** Microbial metabolism of flavonoids through fermentation process.

Substrate	Production	Microorganism	Reference
**Glycosylation**			
Quercetin	Isoquercetin (quercetin-3-glucoside)	*Bacillus cereus*	[63]
Catechin	Catechin 7-α-d-glucopyranoside	*Bacillus stearothermophilus*	[64]
	Catechin 5-α-d-glucopyranoside		
Luteolin	Luteolin-3'-*O*-α-d-glucopyranoside	*Leuconostoc mesenteroides*	[65]
	Luteolin-4'-*O*-α-d-glucopyranoside		
Catechin	Catechin-4'-β-d-fucopyranoside	*Aspergillus niger*	[64]
Kaempferol	Kaempferol 3-β-*O*-glucopyranoside	*Cunninghamella blakesleeana*	[66]
	Kaempferol 4'-*O*-α-L-rhamnopyranoside		
Kaempferol	Kaempferol 3-β-*O*-glucopyranoside	*Cunninghamella echinulata*	[67]
Flavonol	Flavonol 3-β-*O*-glucopyranoside	*Cunninghamella echinulata*	[67]
Quercetin	Quercetin 3-*O*-β-d-glucopyranoside	*Cunninghamella elegans*	[68]
Quercetin	Quercetin glycoside	*Penicillium decumbens*	[69]
Kaempferol	Kaempferol glycoside	*Penicillium decumbens*	[69]
Isorhamnetin	Isorhamnetin glycoside	*Penicillium decumbens*	[69]
**Deglycosylation**			
Daidzin	Daidzein	*Bacillus pumilus*	[31]
Daidzin	Daidzein	*Bacillus subtilis*	[33]
Kaempferol-3-*O*-glucoside	Kaempferol	*Bifidobacterium pseudocatenulatum*	[70]
Naringin	Prunin	*Clostridium stercorarium*	[71]
Quercetin-glucoside	Quercetin	*Lactobacillus plantarum*	[72]
Ploridzin	Phloretin	*Lactobacillus plantarum*	[72]
Kaempferol-3-rutinoside	Kaempferol, kaempferol-3-glucoside	*Aspergillus awamori*	[17]
Rutin	Quercetin, quercetin-3-*O*-glucoside	*Aspergillus awamori*	[17]
Rutin	Quercetin, quercetin-3-*O*-glucoside	*Aspergillus niger*	[73]
Daidzin, glycitin, genistin	Daidzein, glycitein, genistein	*Aspergillus oryzae*	[15]
Narigin	Naringenin	*Curvularia lunata*	[74]
**Ring cleavage**			
Quercetin	2-Protocatechuoylphloroglucinol carboxylic acid	*Aspergillus flavus*	[2]
Flavanone	2'-Hydroxydibenzoylmethane	*Aspergillus niger*	[2]
Quercetin	2-Protocatechuoylphloroglucinol carboxylic acid	*Asperillus niger*	[75]
Flavanone	2'-Hydroxychalcone; 2',4-hydroxydihydrochalcone	*Gibberella fujikuroi*	[76]
	2,4-Dihydroxychalcone		
Flavanone	2',3'',4''-Trihydroxydihydrochalcone	*Penicillium chrysogenum*	[2]
	2'-Hydroxydihydrochalcone		
**Methylation**			
Quercetin	3'-*O*-methylquercetin	*Beauveria* sp.	[77]
Quercetin	Methylquercetin	*Beauveria bassiana*	[78]
Rutin	Methylrutin	*Cunninghamella echinulata*	[78]
Quercetin 3-*O*-β-d-glucopyranoside	Isorhamnetin 3-*O*-β-d-glucopyranoside	*Cunninghamella elegans*	[68]
7-Hydroxyflavanone	7-Methoxyflavanone	*Penicillium chermesinum*	[79]
	3',4'-Dihydroxy-7-methoxyflavanone		
**Glucuronidation**			
Quercetin	Quercetin glucuronide	*Beauveria bassiana*	[78]
Rutin	Rutin glucuronide	*Cunninghamella echinulata*	[78]
Quercetin	Quercetin-4'-*O*-β-d-glucuronide	*Streptomyces* sp.	[20]
	Quercetin-3'-*O*-β-d-glucuronide		
	Quercetin-3-*O*-β-d-glucuronide		
	Quercetin-7-*O*-β-d-glucuronide		
Rutin	Quercetin-4'-*O*-β-d-glucuronide	*Streptomyces* sp.	[20]
	Quercetin-3-*O*-β-d-glucuronide		
Naringenin	Quercetin-7-*O*-β-d-glucuronide		
	Naringenin-7-*O*-β-d-glucuronide	*Streptomyces* sp.	[20]
	Naringenin-4'-*O*-β-d-glucuronide		
**Sulfate conjugation**			
Kaempferol	Kaempferol-4'-sulfate	*Cunninghamella blakesleeana*	[66]
Rutin	Rutin sulfate	*Cunninghamella echinulata*	[78]
Hesperitin	Hesperetin-7-sulfate	*Mucor ramannianus*	[80]
5-Hydroxyflavone	5,4'-Dihydroxyflavone-4'-sulfate	*Streptomyces fulvissimus*	[2]

### 3.1. Glycosylation and Deglycosylation of Flavonoids

#### 3.1.1. Glycosylation of Flavonoids

Glycosylation is the reaction in the biosynthesis of phenolic compounds whereby an activated glycosyl donor is attached to a phenolic aglycone through linkage to hydroxyl groups. This reaction could be performed by glycosyltransferases, resulting in a higher hydrophilic solubility of these, mainly lipophilic compounds. The glycosylation process could be applied to enhance the stabilization, detoxification and solubilization of the substrates [67]. Some microorganisms such as *Bacillus cereus*, *Streptomyces rimosus*, *Cunninghamella elegans* and *Cunninghamella echinulata* are known to be capable of glycosylating phenolic compounds [63,67,68,81]. Quercetin can be converted into isoquercetin (quercetin-3-glucoside) with a 20% bioconversion yield using a fermentation process with *Bacillus cereus* at 30 °C [63]. As reported by Zi *et al.* [68], incubation of quercetin with *Cunninghamella elegans* ATCC9245 yielded quercetin-3-*O*-β-d-glucopyranoside. Similar to quercetin, kaempferol was glycosylated by the filamentous fungus *Cunninghamella echinulata* [67]. According to Slana *et al.* [82], under conditions whereby flavonoids are toxic, e.g., when they are present in high concentrations, microorganisms such as fungi could produce glycosylating enzymes (glycosyltransferase) transforming the phenolic compounds into less toxic metabolites. Not only glycolysltransferases but also other enzymes can be involved in the glycosylation of phenolic compounds, as it was shown e.g., for glucansucrase of *Leuconostoc mesenteroides* acting on luteolin, quercetin and myricetin [65], cellulase of *Penicillium decumbrens* on quercetin [69], cellulase of *Aspergillus niger* on catechin [64], or α-amylase of *Bacillus* sp. on catechin [64].

#### 3.1.2. Deglycosylation of Flavonoids

In contrast to glycosylation, many studies indicated that deglycosilation of phenolic compounds could be performed through microbial fermentation due to glycosyl hydrolase activities, such as β-glucosidase [28,52], naringinase [74], α-rhamnosidase and hesperidinase [17]. Park *et al.* [83] reported that glucose moieties attached to flavonoids at the C3 and C7 positions can be a substrate for β-glucosidase [EC 3.2.1.21]. This enzyme is well known for its deglycosylation capability by hydrolyzing the β-1,4 glycosidic bonds in aryl and alkyl β-d-glucosides as well as glycosides containing disaccharides and oligosaccharides [52,84]. Recently, several studies for increasing the concentration of isoflavone aglycones in soy products have been performed [15,31,70]. Cho *et al.* [31,33] reported that fermented soybeans with *Bacillus pumilus* HY1 or *Bacillus subtilis* CS90 for 48 h incubation resulted in the highest concentration of isoflavone aglycones (daidzein) and thus in a decrease in isoflavone glucosides. This finding was also reported by Lee *et al.* [15], who found that soybeans fermentation by *Aspergillus oryzae* KACC 40247 seemed to cause a significant increase in the amount of isoflavone aglycones including daidzein, glycitein and genistein. In general, the biotransformation of glycosidic flavonoids occurring in soybeans into their corresponding aglycones during fermentation was attributed to microbial β-glucosidase activity [15,31,33]. This enzyme could be considered as a possible reason for the deglycosylation of kaempferol-3-*O*-glucoside into kaempferol through fermentation with *Bifidobacterium pseudocatenulatum* B7003 [70] or *Aspergillus awamori* [17], quercetin-3-glucoside into quercetin by *Aspergillus awamori* [17]. 

Another enzyme also produced by fungal fermentation is α-l-rhamnosidase [EC 3.2.1.40], which cleaves terminal α-l-rhamnose present in many natural glycosides such as naringin, rutin, quercitrin, hesperidin, diosgene, terpenyl glycosides [85]. For example, fermentation of rutin with food-grade *Aspergillus niger* for 4 h [73] or *Aspergillus awamori* for 4 days [17] resulted in transformation of rutin into (isoquercetin) quercetin-3-glucoside which could be attributed to α-l-rhamnosidase activity well known for its capability of removing one rhamnose moiety. Also some α-rhamnosidase activity was measured in different lactic acid bacteria, with the most positive strains belonging to *Lactobacillus plantarum* and able to release rhamnose units from hesperidin and rutin rhamnose metabolites [86].

### 3.2. Ring Cleavage of Flavonoids

Many flavonoids undergo a ring-opening reaction in which their C-ring is split and chalcones along with hydroxylations at different C-positions are produced. As shown by Udupa *et al.* [76], a number of hydroxylated chalcone metabolites (2'-hydroxychalcone; 2',4-dihydroxydihydrochalcone; 2',4-dihydroxychalcone) are produced when flavanone was incubated with the fungal strain, *Gibberella fujikuroi*. The ring fission of the heterocylic C-ring of flavanone is known to occur among several fungal species such as *Aspergillus*, *Penicillium*, *Rhizopus*, *Monacus* [2]. Incubation of unsubstituted flavanone with *Aspergillus niger* x172 yielded the chalcone products 2'-hydroxydibenzoylmethane and 2',3'',4''-trihydroxydihydrochalcone [2]. Another strain, *Penicillium chrysogenum* cleaves the C-ring of flavanone into 2'-hydroxydihydrochalcone [2].

Similarly, biotransformation of quercetin to 2-protocatechuoylphloroglucinol carboxylic acid was observed by Das *et al.* [2], who indicated that the C-ring of quercetin was oxidized and cleaved by the enzyme quercetinase produced by *Aspergillus flavus*. The cleavage of quercetin to 2-protocatechuoylphloroglucinol carboxylic acid was also performed by flavonol 2,4-dioxygenase, an enzyme produce by *Aspergillus niger* DSM 821 [75]. 

### 3.3. Methylation of Flavonoids

*O*-Methylated flavonoids, known as xenobiotic transformation metabolites, is a common hepatic metabolite obtained by phase II reaction occurring in mammalians by *O*-methyl transferases [1]. However, some fungal species have been evaluated for their capability of methyl conjugation [68,77,78,79]. According to Eula Maria de *et al.* [77], some of *Beauveria* strains used in their study exhibited the ability to produce 3'-*O*-methylquercetin by incubation of quercetin at 29 °C for 72 h. Another flavonoid, rutin was also methylated into methylrutin by *Cunninghamella echinulata* [78]. The fermentation of 7-hydroxyflavanone with *Penicillum chermesinum* 113 at 25 °C for six days resulted in two methylated products: 7-methoxyflavanone and 3',4'-dihydroxy-7-methoxyflavanone [79]. *O*-Methylation was also found in the transformation pathway of quercetin into isorhamnetin 3-*O*-β-d-glucopyranoside as reported by Zi *et al.* [68] using *Cunninghamella elegans* ATCC 9245 at 28 °C for 72 h.

### 3.4. Glucuronidation of Flavonoids

The use of metabolism of bioactive compounds with microorganisms to produce specific mammalian metabolites started a decade ago. A few microbial strains are known for their ability to produce flavonoid metabolites by glucuronidation such as *Beauveria bassiana* [78], *Cunninghamella echinulata* [78] and *Streptomyces* sp. [20]. Araújo *et al.* [78] reported that fermentation of quercetin and rutin with *Beauveria bassiana* and *Cunninghamella echinulata* respectively resulted in their corresponding glucuronide. Recently, a study has demonstrated that a fermentation process of several phenolic compounds (e.g., naringenin, rutin, quercetin) using a *Streptomyces* sp. can produce glucuronidated products [20]. According to these authors, quercetin incubated with a culture of *Streptomyces* M52104 at 28 °C for 65 h resulted in several glucuronidated compounds including quercetin-4'-*O*-β-d-glucuronide, quercetin-3-*O*-β-d-glucuronide and quercetin-7-*O*-β-d-glucuronide. Similarly, both naringenin and naringenin-7-*O*-glucoside were also glucuronidated into naringenin-7-*O*-β-d-glucuronide and naringenin-4'-*O*-β-d-glucuronide by fermentation with *Streptomyces* M52104 at 28 °C for 65 h [20]. The microbial production of glucuronidates could be attributed to the detoxification pathways in which bioactive compounds are conjugated with glucuronic acid leading to an increased solubility and a higher molecular weight [20].

### 3.5. Sulfate Conjugation of Flavonoids

Sulfate conjugation is a major pathway for the phase II metabolism of phenolic compounds in humans via the bile using arylsulphotransferase, originating from human colonic bacteria [1]. However, recent studies have shown that bioconversion of phenolic compounds into their sulfated conjugated form could also be performed by a few fungal strains including *Cunninghamella echinulata* [78], *Cunninghamella blakesleeana* [66], *Streptomyces fulvissimus* [2], and *Mucor ramannianus* [80]. Rutin incubated with *Cunninghamella echinulata* induced rutin sulfate [78]. 5-Hydroxyflavone was converted into 5,4'-dihydroxyflavone-4'-sulfate by *Streptomyces fulvissimus* [2]. Ibrahim *et al.* [66] reported that incubation of kaempferol with *Cunninghamella blakesleeana* (ATCC 8688A) led to the production of kaempferol-4'-sulfate. A similar result was observed by Herath *et al.* [80] indicating that *Mucor ramannianus* (ATCC 2628) was able to convert hesperitin into hesperetin-7-sulfate. 

## 4. Perspectives

From the previous sections, it becomes clear that different microorganisms can produce a whole range of metabolites. To our view there will probably be many more conversion products formed in nature, than the ones we presented here, especially as we excluded natural fermentation processes in the discussion of this review. Natural fermentation processes contain an enormous diversity in microflora, often uncharacterized, but also the potential of these microorganisms towards the metabolism of secondary plant compounds are unknown. Even with controlled fermentation processes, using well-defined microorganisms, there is a lack of knowledge on the conversion potential of phenolic compounds. So, more studies in this area are needed to elucidate the microbial pathways of flavonoid conversion, identification of the metabolites, and bioactivity determinations. However research to answer these research gaps is often hampered by the lack of flavonoid standards in quantities large enough to carry out the fermentation experiments. Of course one could use well-defined plant extracts as sources of flavonoids in the fermentation processes, but it is known that additive, synergistic and antagonistic effects exist between phenolic compounds and thus would effect the conversions. A lot of studies have been done using plant extracts, however the extraction yield and profile of the phenolic compounds can differ between extraction batches due to the high variability of the phenolic compounds present in the raw plant material. Also a detailed identification and quantification of the phenolic compounds of these extracts are often lacking, although it is necessary information. Indeed analytical tools to identify and further quantify the metabolites becomes very specialized, but crucial to understanding the microbial metabolism of flavonoids. Furthermore, in depth insight in the reasons for the conversion of flavonoids by microorganisms is really needed. Some results point out that this conversion is a detoxification mechanism, but toxic levels and mechanisms of toxicity of the newly formed metabolites are unknown. This insight will allow us to go back to the microbial metabolic pathways, giving essential information needed for the development of genetically modified microorganisms overproducing specific metabolites. However, overproduction of metabolites is only relevant if these conversion products exert a (high and/or interesting) bioactivity. Several studies are done showing antioxidative and antimicrobial activity of the aglycone flavonoid form. Only few studies have dealt with their inhibitory effect on (digestive) enzymes, coloring effects, other health effects (e.g., antihypertensive, anticarcinogenic, anti-obesity), or their function as an herbicide or insecticide. However, studies evaluating the bioactivities of microbial flavonoid metabolites (e.g., sulfated compounds, hydroxylated compounds) are rare. Therefore, more studies dealing with this research gap can lead to the discovery of a huge potential of new bioactive natural compounds.

## 5. Conclusions

In summary, increased release of phenolic compounds in fermented plant-based foods is due to the action of cell wall-degrading enzymes produced through fermentation. In addition, microbial fermentation can induce the bioconversion of flavonoids into their metabolites by different pathways including glycosylation, deglycosylation, ring cleavage, methylation, glucuronidation and sulfate conjugation according to microbial strains and substrates. Thus, microbial fermentation could be considered as a potential technology for releasing phenolic compounds from natural resources, as well as for producing new bioactive compounds. Although some fermentation processes are applied in the production of phenolic compounds, the yield of bioconversion is variable, depending on the fermentation parameters used (e.g., microorganisms, medium, temperature, pH) as well as due to differences in the plant matrix itself. Therefore, further research into optimal process is required. Also, there is still a lack of knowledge on the metabolic pathways as well as the relationship between specific metabolites and their corresponding bioactivity, bioaccessibility and bioavailability, which thus demands more research in this field.
